# Biomarker and Translational Prostate Cancer Research

**DOI:** 10.1155/2015/309851

**Published:** 2015-01-15

**Authors:** Andreas Doll, Jeremy Clark, Colleen Nelson

**Affiliations:** ^1^Biomedical and Translational Oncology Research Unit, Institut de Recerca Vall d'Hebron, Edifici Collserola, Passeig de la Vall d'Hebron, 119-129 Barcelona, Spain; ^2^Cancer Genetics, University of East Anglia, Norwich, Norfolk NR4 s7TJ, UK; ^3^Australian Prostate Cancer Research Centre, Brisbane, QLD 4102, Australia

The existing clinical biomarkers for prostate cancer (PCa) are not ideal, since they cannot specifically differentiate between those patients who should be treated immediately and those who should avoid overtreatment. Current screening techniques lack specificity, and a decisive diagnosis of PCa is based on prostate biopsy. Although PCa screening is widely utilized nowadays, two-thirds of the biopsies performed are still unnecessary. Thus, the discovery of noninvasive PCa biomarkers remains an urgent unmet medical need. Once metastasized, there is still no curative therapy. A better understanding of sustained androgen receptor signalling in castration resistant prostate cancer (CRPC) has now led to the development of more effective therapies. We need a better understanding of the molecular and cellular aspects of prostate carcinogenesis and progression. Identification of cancer initiating cells and therapies against these populations is a promising way forward to fight this disease.

